# Multi-Elemental Analysis as a Tool to Ascertain the Safety and the Origin of Beehive Products: Development, Validation, and Application of an ICP-MS Method on Four Unifloral Honeys Produced in Sardinia, Italy

**DOI:** 10.3390/molecules27062009

**Published:** 2022-03-21

**Authors:** Andrea Mara, Sara Deidda, Marco Caredda, Marco Ciulu, Mario Deroma, Emanuele Farinini, Ignazio Floris, Ilaria Langasco, Riccardo Leardi, Maria I. Pilo, Nadia Spano, Gavino Sanna

**Affiliations:** 1 Dipartimento di Chimica e Farmacia, Università degli Studi di Sassari, Via Vienna 2, I-07100 Sassari, Italy; a.mara@studenti.uniss.it (A.M.); saradeidda96@tiscali.it (S.D.); marcociulu@yahoo.it (M.C.); ilangasco@uniss.it (I.L.); mpilo@uniss.it (M.I.P.); nspano@uniss.it (N.S.); 2 AGRIS Sardegna, Loc. Bonassai S.S. 291 Km 18.6, I-07100 Sassari, Italy; mcaredda@agrisricerca.it; 3 Dipartimento di Agraria, Università degli Studi di Sassari, Viale Italia 39/a, I-07100 Sassari, Italy; mderoma@uniss.it (M.D.); ifloris@uniss.it (I.F.); 4 Dipartimento di Farmacia, Università di Genova, Viale Cembrano 4, I-16148 Genova, Italy; farinini@difar.unige.it (E.F.); leardi@difar.unige.it (R.L.)

**Keywords:** honey, ICP-MS, trace elements, toxic elements, botanical origin, geographical origin, asphodel, eucalyptus, strawberry tree, thistle

## Abstract

Despite unifloral honeys from Sardinia, Italy, being appreciated worldwide for their peculiar organoleptic features, their elemental signature has only partly been investigated. Hence, the principal aim of this study was to measure the concentration of trace and toxic elements (i.e., Ag, As, Ba, Be, Bi, Cd, Co, Cr, Cu, Fe, Hg, Li, Mn, Mo, Ni, Pb, Sb, Sn, Sr, Te, Tl, V, and Zn) in four unifloral honeys produced in Sardinia. For this purpose, an original ICP-MS method was developed, fully validated, and applied on unifloral honeys from asphodel, eucalyptus, strawberry tree, and thistle. Particular attention was paid to the method’s development: factorial design was applied for the optimization of the acid microwave digestion, whereas the instrumental parameters were tuned to minimize the polyatomic interferences. Most of the analytes’ concentration ranged between the relevant LoDs and few mg kg^−1^, while toxic elements were present in negligible amounts. The elemental signatures of asphodel and thistle honeys were measured for the first time, whereas those of eucalyptus and strawberry tree honeys suggested a geographical differentiation if compared with the literature. Chemometric analysis allowed for the botanical discrimination of honeys through their elemental signature, whereas linear discriminant analysis provided an accuracy level of 87.1%.

## 1. Introduction

Honey is an ancient natural and functional food used for centuries in the traditional medicine of many cultures. Beyond monosaccharides, such as glucose and fructose, which it is composed of up to 80% *w/w*, honey is a very complex matrix containing many bioactive compounds such as proteins, amino acids and enzymes, organic acids, polyphenols, flavonoids, vitamins, and inorganic elements [1]. This combination makes honey an outstanding functional food with many health-promoting activities and antimicrobial, antiviral, antifungal, anticancer, and antidiabetic properties [2,3,4,5,6].

Sardinia, Italy, is the second-largest island in the Mediterranean Sea. Its extension and distance from the European and African continental shelves characterize the endemic flora and fauna. Furthermore, due to the paucity of population and industries, the poor anthropogenic pressure is considered by consumers as an intrinsic guarantee of the quality of agri-food products, albeit sometimes this is not supported enough by scientific data. For these reasons, Sardinian honey is a typical product universally appreciated and recognized for its quality and for its peculiar organoleptic features.

Beekeeping is a significant sector of the regional agriculture, since Sardinia produces more than the 11% of Italian honey, and Italy is the fourth largest producer in the European Union [7]. Besides the multiflora ones, the production of Sardinian honey is mainly focused on four unifloral varieties: asphodel (*Asphodelus* spp.), eucalyptus (*Eucalyptus* spp.), strawberry tree (*Arbutus unedo* L.), and thistle (*Galactites tomentosa*) [8,9].

Given the importance of the sector for the economy of Sardinia, the traceability of their unifloral honeys should be ascertained with analytical methods aimed toward food authentication. Among the possible methods, those based on elemental metabolomics [10] provide great achievements in both botanical [11,12] and geographical classification [11,13]. The elemental metabolomics approach involves the determination of trace elements to achieve an elemental signature [10]. Hence, although they are not directly related with most of the therapeutical and nutraceutical properties of honey [14,15,16,17], trace elements are nevertheless of great scientific interest.

First, the health-promoting properties of honey must be coupled with the highest level of food safety and, consequently, the concentration of toxic elements should be negligible. Thus, honey is considered a valuable bioindicator of environmental pollution [18,19,20,21] because its chemical composition is strictly related to the environmental quality of the area next to the beehive [22,23]. In fact, the concentration of some potentially toxic elements has been found to be higher in hive matrices from industrial and urban areas with respect to what was measured in uncontaminated areas [18,24,25,26,27]. For these reasons, the concern for the potential presence of toxic elements in honey moved the European Union to set maximum levels for Hg (0.01 mg kg^−1^) [28] and for Pb (0.1 mg kg^−1^) [29].

Finally, the elemental signature of honey has been frequently used for its authentication and traceability because foraged plants tend to accumulate specific elements in the nectar, allowing for classification according to their botanical origin [30,31]. In addition, soil composition affects element availability in honey, allowing, in this case, their georeferentiation [31,32].

Despite trace elements being determined in honeys from many countries, such as Bulgaria [12], Kosovo [20], Ethiopia [24], Turkey [26], Italy [18,27,32,33], Poland and Greece [31], Hungary [34], and New Zealand [35], there are few contributions to the literature reporting on the amounts of trace elements in unifloral honeys produced in Sardinia. The literature is also poor regarding studies reporting on the elemental composition of the honeys of asphodel, eucalyptus, strawberry tree, and thistle produced out of Sardinia. Among them, eucalyptus honey was the most investigated [36,37,38,39] and data from Tunisia [36], Argentina [37], and Italy [38] have been reported in the literature. In contrast, the elemental composition of strawberry tree honey has been reported only for those coming from Croatia [40], whereas, to the best of our knowledge, no study has dealt with a trace element characterization of either asphodel or thistle honeys.

From an analytical viewpoint, microwave-assisted digestion [41,42] and inductively coupled plasma spectrometry (ICP-MS) [41,43] are the preferred techniques for sample pre-treatment and for elemental analysis, respectively. Among the former ones, microwave-assisted digestion ensures high efficiency and good performances [44,45,46,47]. On the other hand, ICP-MS allows to achieve great results in trace and ultra-trace analysis and to perform a reliable investigation for food authentication and traceability [13,41]. Regardless of the tool chosen for the elemental analysis, the data obtained should be elaborated using a multivariate approach, essential for classification purposes. Principal component analysis (PCA) [36], cluster analysis (CA) [32], discriminant analysis (DA) [35], partial least squares (PLS) [31], and self-organizing maps (SOMs) [12] provide great results in exploratory analysis and honeys classification.

This research group has been active for decades in the assessment and validation of original analytical methods applied to beehive matrices to ascertain their quality [48,49,50,51,52,53,54,55] as well as origin [56,57]. Hence, the principal aim of this research was to ascertain the elemental signature of the most renowned unifloral honeys of Sardinia as a reliable tool to ensure their healthiness and guarantee their origin. Therefore, an original ICP-MS method able to simultaneously measure the total amount of 23 elements of potential health concern (i.e., Ag, As, Ba, Be, Bi, Cd, Co, Cr, Cu, Fe, Hg, Li, Mn, Mo, Ni, Pb, Sb, Sn, Sr, Te, Tl, V, and Zn) was developed and thoroughly validated. Finally, the proposed method was applied to a large sampling of honeys from asphodel, eucalyptus, strawberry tree, and thistle.

## 2. Results and Discussion

### 2.1. Sample Pre-Treatment

To avoid the matrix effect and maximize the signal-to-noise ratio, great attention was paid to the optimization of the sample pre-treatment step [44,45,46,47]. Although the sample amounts and the instrumental conditions depend on the specific features of the microwave system, according to the recent method proposed by Astolfi et al. [42], HNO_3_ and H_2_O_2_ were chosen as the acidic/oxidizing mixture due the fact of their proven capability to minimize the formation of the interfering polyatomic ions in the plasma and their efficiency in organic matter decomposition. According to Muller et al. [45], a simply 2^2^ full factorial design was applied to improve the digestion efficiency and the composition of the oxidizing mixture. The optimization allowed for a reduction in the HNO_3_ volume required for sample digestion, increasing the amount of the greenest hydrogen peroxide and the mass of sample digested, which is an important achievement for trace element analysis. These results were also achieved thanks to the versatile performance of the ultraWAVE digestion system. The optimized method is reported in Table 1.

First, honey samples were heated until 40 °C and then homogenized by an Ultraturrax. Next, approximately 0.7 g of honey, exactly weighted on an analytical balance (±0.0001 g uncertainty), was treated with 0.5 cm^3^ of HNO_3_, 3 cm^3^ of H_2_O_2_, and 4 cm^3^ of type I water inside a PTFE vessel of the SCR mineralization system. After the digestion, samples were diluted to a final volume of 15 cm^3^ and filtered through a 0.22 μm nylon filter. Finally, they were stored at 4 °C in the dark until the analysis. The typical amount of residual acidity found in digested samples was 0.2 mol dm^−3^, whereas the efficiency of organic matter decomposition (EOMD%) was generally higher than 94%.

### 2.2. Validation of the ICP-MS Method

The validation of the ICP-MS method proposed was accomplished in terms of the limit of detection (LoD), limit of quantification (LoQ), linearity, precision, and trueness. Table 2 reports the validation parameters of the ICP-MS method for the determination of the total amount of 23 trace elements in honey.

The LoDs and LoQs of the method were calculated according to Currie [58] on 30 measurements of method blanks obtained in different analytical sessions. The LoDs were generally below 10 μg dm^−3^ for all of the elements except for Ba, Cu, Fe, and Zn, which were between 10 and 40 μg dm^−3^.

Because of the large variability of the analyte concentrations, great attention was paid to the instrument calibration phase. Although the linearity of the ICP-MS could be extended over several orders of magnitude of concentration, in this case, this parameter was explored for each element only within the relevant operative range of concentration. For each analyte, the calibration function was the result of a linear regression of six standard solutions, three for each extreme point of the calibration range. To evaluate the experimental error and verify the linearity of the calibration function, three different solutions at an analyte concentration equal to the central point of the calibration range were analyzed. For each analyte, the difference between the experimental and predicted values was not significant (*t*-test, α = 0.05). Hence, keeping in consideration the random distribution of the residuals around the mean value as well as the very high coefficient of determination (*R*^2^ always above 0.999), the linearity of the calibration function was successfully ascertained.

Precision, measured as the coefficient of variation (CV%), was assessed in terms of repeatability and intermediate precision. For this purpose, a batch of honey samples was replicated in the same way and in different analytical sessions. Table 2 reports the CV%s calculated at the analyte’s average concentration. For elements in which the concentration in the honey was below the LoD, precision was measured by spiking the samples with standard solutions of analyte. Repeatability exhibited CV% between 1% (Ba) and 12% (Zn), while intermediate precision ranged between 3% (Ba and Mn) and 21% (Te). All precision parameters were in the range of CV% defined by the Horwitz’s theory [59]; hence, the overall precision level of the method was acceptable.

Due to the lack of certified reference material (CRM) for the determination of trace elements in honey, trueness was evaluated by recovery tests. Hence, three aliquots of each sample were spiked with increasing amounts of a standard solution containing all analytes. All aliquots then underwent the whole analytical procedure described in Section 2.1. Furthermore, an additional aliquot for each honey sample was analyzed without any spiking. Recoveries ranged between 85% (Bi) and 130% (Hg). Quantitative recoveries (*t*-test, α = 0.05) were observed for most of the elements, except for As, Li, Mo, Pb, and V (underestimation bias) and for Be, Cd, and Sb (overestimation bias). Nevertheless, the recoveries obtained in these cases were also acceptable according to the AOAC guidelines [60]. In summary, the proposed method was fully and successfully validated, and the parameters here considered were comparable or better than those reported in previous works [31,35,36,41,42].

### 2.3. Honey Analysis

Table 3 summarizes, in terms of both the mean value and range, the amount of toxic and trace elements belonging to the four unifloral Sardinian honeys, while the full data set is available in the Supplementary Materials (Table S1).

Table 4 shows the results of the amounts of toxic and trace elements reported for unifloral eucalyptus honeys produced in different countries.

The determination of As, Cd, Cr, Cu, Fe, Mn, Ni, Pb, and Zn was performed in all studies, and the amounts of these elements in honeys from different geographical origins normally spanned over one or two orders of magnitude. The different concentrations of the most abundant elements (i.e., Cr, Cu, Fe, Ni, Pb, and Zn, significantly higher in the samples from Tunisia, or the very high concentration of Mn in the Argentinian honey) allowed to envisage the feasibility of a discrimination of these honeys according to geographical origin. However, due to the paucity of the samples analyzed in the literature [36,37,38,39], no definitive conclusion could be obtained by this comparison.

**Figure 1:**
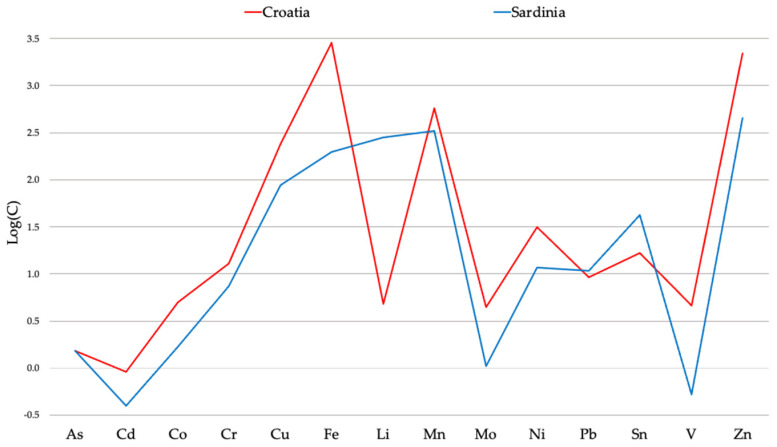
Average distributions of strawberry tree honeys from Croatia and Sardinia. Elemental concentrations are in μg kg^−1^.

The comparison of the data set obtained from the determination of 23 elements in nine samples of strawberry tree from Croatia [40] was more reliable. In this case, 17 out of 23 elements were measured in both countries. Meaningful differences in the average concentrations of many trace elements (i.e., Co, Cu, Mo, Ni, Pb, Sb, Sn, and V) appeared to support the possibility to achieve a differentiation according to the different geographical origin. The average distributions reported in Figure 1 highlights the differences between Croatian and Sardinian strawberry tree honeys.

### 2.4. Chemometric Analysis

In order to evaluate the suitability of trace elements to ascertain the origin of Sardinian honeys, principal component analysis (PCA) and linear discriminant analysis (LDA) were performed for exploratory and classification purposes, respectively.

The original data set, constituted by 133 samples and 18 variables (Ag, Be, Hg, Sb, and Te were removed because they were almost never quantified) were randomly divided into training (85 samples) and validation (48 samples) sets for internal validation.

The logarithmic transformation (log10) was used to reduce the skewness of the probability density distribution present in the original data. However, the information embodied in the loadings has changed and so it could lead to dangerous misunderstandings [61].

**Figure 2:**
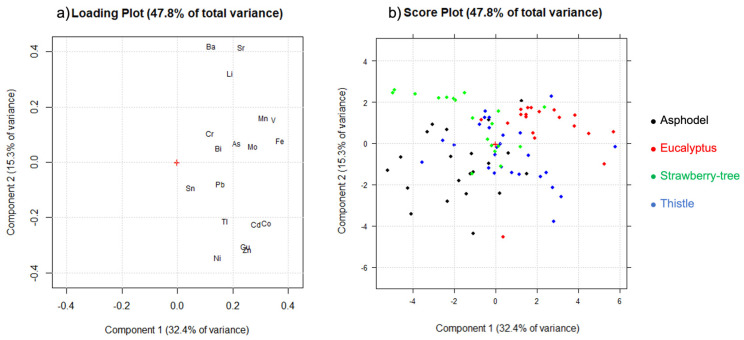
PCA performed on 85 unifloral honey samples and 18 trace elements: (**a**) loading plot; (**b**) score plot. Objects were colored according to the different botanical origin of the samples.

From the loading plot (Figure 2a), PC1 explained mainly the global concentration of the trace elements characterized by positive loadings on PC1 (Fe, V, Mo, and Mn). On the other hand, PC2 explained the contrast between the two clusters of correlated trace elements: Li, Sr, and Ba at positive loadings and Co, Cd, Cu, Zn, and Ni at negative loadings. Couples of elements, such as Mn and V, Cd and Co, and, mainly, Cu and Zn, were strongly correlated among them. Finally, Sn, Cr, Pb, and Bi were the less significant variables, as suggested by their overall low expression in both PC1 and PC2. Looking at the score plot in the plane PC1–PC2 (Figure 2b), with the objects colored according to the different botanical origin, it was noticed that the four classes were generally characterized by a specific location on the plane: asphodel honey samples (1) can be found at negative scores of both PC1 and PC2; eucalyptus samples (2) at positive scores of both PC1 and PC2; strawberry tree samples (3) at negative score of PC1 and positive scores of PC2 and, finally, thistle samples (4) can be found at positive scores of PC1 and negative scores of PC2. The information on the within-class variability of the four classes was smoothed by the logarithmic transformation, as well as the correlations between the variables. The high variability can be confirmed from the width of the ranges reported in Table 3. Furthermore, an analysis of the correlations was performed before and after the logarithmic transformation (Table S2) to ensure a correct interpretation of the variables. The coefficients highlight a strong correlation between Fe–Mn–V and Cu–Ni–Zn, confirmed by the loading plot, in contrast, the correlations between Ba–Sr–Li and Cd–Co were emphasized by the data pre-treatment.

For classification purposes, linear discriminant analysis (LDA) was used. This method is based on the description of data by means of probability density distributions under two hypotheses: (i) probability distributions are multivariate normal within all the classes; (ii) dispersion and correlation structures are the same within all the classes. Fortunately, the method is quite robust against slight deviations from these hypotheses [62].

In this case, after the pre-processing, it was possible to use LDA because the classes were similar in size and orientation and, therefore, it was possible to assume that they had similar variance and covariance matrix.

The results obtained in cross-validation and prediction are reported in Table 5. The botanical origin with the lowest percentage of correct prediction was asphodel (61.9%), whose samples were mainly confused with thistle (*n* = 3) and strawberry tree (*n* = 5), while the best results were obtained for eucalyptus (84.2%). A permutation test was performed to compare the performance obtained with the correct assignment with a series of random permutation of the classes (in this case, 100 permutations were calculated). Figure S3 shows how the model works, since in no event in the classification were the results obtained randomly. In prediction, a correct classification percentage of 87.1% was achieved, quite in agreement with the results obtained in the CV. By observing the confusion matrix (Table 6), it is possible to look at the samples characterized by the wrong assignment, highlighting that asphodel and thistle were relatively less accurate but still acceptable: strawberry tree was twice erroneously classified as thistle and once as asphodel; eucalyptus was erroneously re-classified as both thistle and asphodel; a sample of asphodel was wrongly classified as a thistle.

The level of discrimination achieved in this contribution is less than those obtained by this research group using chemometric approaches based on untargeted physical and chemical data [49] or FT-ATR spectroscopic data [50].

Several factors could affect the ascertainment of the botanical origin. First, Sardinian unifloral honeys are typically underrepresented from a melissopalynological point of view since the pollen spectrum is often largely overlapping due to both accompanying and rare pollens [8]. This highlights a partly common botanical origin, especially for spring productions of asphodel and thistle and, secondly, also for summer and autumn production of eucalyptus and strawberry tree, respectively. Finally, a last possible factor, could be related to the geographical origin of some honeys, especially for those coming from the mining areas (i.e., central and southwestern Sardinia), where the natural characteristics of soil composition may have influenced the concentration of some elements.

In addition, a second LDA was unsuccessfully used in the attempt to achieve a geographical classification of Sardinian samples belonging to the same floral origin (data not reported). Probably, the reason for this failure was related both to the difficulty to localize the exact position of the hives [8] and to the extreme geochemical and pedological variability of the soil of Sardinia [63].

Summarizing, the elemental signature provided rather robust descriptors for the botanical discrimination of Sardinian unifloral honeys, whereas the intra-regional geographical discrimination was currently compromised and should be investigated by acquiring additional data. Hence, efforts will be paid to carefully solve these issues. Moreover, additional samples of both eucalyptus and strawberry tree honeys produced outside Sardinia will be gathered in order to achieve a reliable geographical discrimination.

## 3. Materials and Methods

### 3.1. Honey Samples

**Figure 3:**
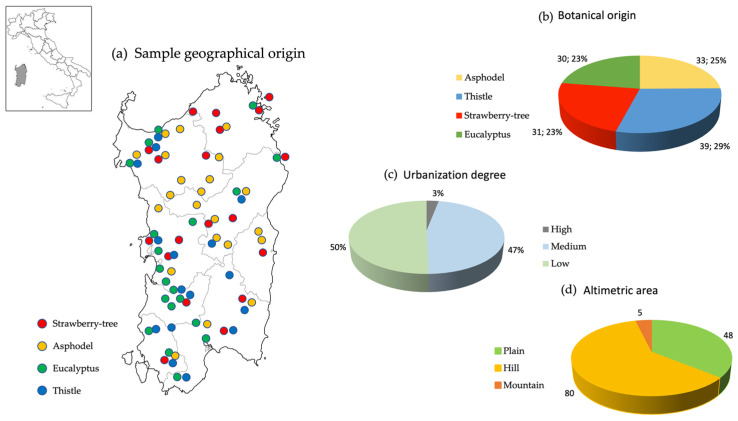
Information about Sardinian honey collection (*n* = 133). (**a**) Sample geographical origin; (**b**) Botanic source; (**c**) Urbanization degree; (**d**) Altimetric area.

The honeys were mainly gathered in the last year directly from local beekeepers and, in a minor amount, purchased in Sardinian markets. The collection, summarized in Figure 3, consisted of 133 samples of the four most renowned unifloral varieties of honey: asphodel (*n* = 33), eucalyptus (*n* = 30), strawberry tree (*n* = 31), and thistle (*n* = 39). Samples were stored in the dark at 4 °C until the analysis. The botanical origin of each sample, primarily based on information directly provided by beekeepers, has been confirmed by the melissopalynological analysis, which provided data within the relevant ranges measured by Floris et al. [8].

Briefly, Sardinia is mainly hilly, therefore, 60% (*n* = 80) of the honeys are from the hills, 36% (*n* = 48) from the plains, and only 4% (*n* = 5) from the mountains. The production areas are divided into rural areas 50% (low urbanization degree, *n* = 67), slightly urbanized areas 45% (medium urbanization degree, *n* = 62) and urbanized 3% (high urbanization degree, *n* = 4) [64]. The odd distribution of the samples in the region, as reported in Figure 3, is representative of the different production areas and the distribution of the botanical sources.

### 3.2. Reagents and Standard Solutions

In all the analytical phases, type I water (resistivity > 18 MΩ cm^−1^), produced by means of a MilliQ plus System (Millipore, Vimodrone, Italy) was used. Nitric acid (67–69% *w/w*, NORMATON^®^ for ultra-trace analysis) and hydrogen peroxide (30% *w/w*, NORMATON^®^ for ultra-trace metal analysis) were from VWR (Milan, Italy). The multi-element standard periodic table mix 1 for ICP (TraceCert^®^, 33 elements, 10 mg dm^−3^) and the internal standard solution (3% HNO_3_ *v/v* aqueous solution containing 1000 mg dm^−3^ of Rh) were from Sigma-Aldrich (St. Louis, MI, USA), while the elemental standards of Hg (10 mg dm^−3^), Mo (10 mg dm^−3^), Sb (1000 mg dm^−3^), and Sn (100 mg dm^−3^) were from Carlo Erba (Milan, Italy). NexION KED Setup Solution (1% HCl *v/v* aqueous solution containing Co 10 μg dm^−3^ and Ce 1 μg dm^−3^) and NexION Setup Solution (1% HNO_3_ *v/v* aqueous solution containing 1 μg dm^−3^ each of Be, Ce, Fe, In, Li, Mg, Pb, and U) were from Perkin Elmer (Milano, Italy). A standard solution of NaOH 0.5 mol dm^−3^, and the potassium dichromate, the ammonium iron (II) sulphate, and the sulfuric acid (96% *v/v*) used for the titrations were from Sigma-Aldrich.

### 3.3. Instrumentation

The multi-element analysis was performed on a NexION 300X ICP-MS spectrometer, equipped with a S10 autosampler, a glass concentric nebulizer, a glass cyclonic spray chamber, and a kinetic energy discrimination (KED) collision cell, all produced by Perkin Elmer (Milan, Italy). Samples were mineralized by means of a microwave single reaction chamber (SCR) system (ultraWAVE™, Milestone, Sorisole, Italy) equipped with fifteen polytetrafluoroethylene (PTFE) vessels (volume: 15 cm^3^ each). An Ultraturrax mixer model T18 (IKA, Staufen, Germany) was used to homogenize the honey samples before analysis. The residual acidity and the dissolved organic carbon (DOC) in the digested sample were determined by acid–base titration procedure and by the Walkley-Black method [65] using a Thermo Scientific Orion 950 titrator, whereas the total organic carbon (TOC) was determined by means of a CHN analyzer Leco 628. Nylon filters (pore diameter: 0.22 μm), polypropylene (PP) metal-free tubes, and polyethylene (PE) flasks were from VWR (Milan, Italy).

### 3.4. ICP-MS Method Assessment, Quality Control and Assurance

The minimization of the dissolved organic carbon (DOC) in digested honey samples is an essential prerequisite to make a reliable ICP-MS analysis. Failing in this, high residual amounts of organic substances from a partial oxidation of the saccharides contained in honey could cause the formation of molecular ions in the plasma that may interfere with the determination of very important elements such as ^52^Cr, ^63^Cu, and ^75^As. Beyond this specific issue, the need to avoid the interference of molecular ions of any origin on the determination of the chosen isotopes of the analytes suggested to operate in kinetic energy discrimination (KED) mode for 15 elements out of 23. Therefore, the He flow has been carefully optimized for each element, to find the best compromise between the minimization of the polyatomic ion interferences and the maximization of the instrumental signal [66,67,68,69]. As a result, ^75^As, ^138^Ba, ^111^Cd, ^59^Co, ^52^Cr, ^63^Cu, ^57^Fe, ^55^Mn, ^60^Ni, ^121^Sb, ^120^Sn, ^88^Sr, ^120^Te, ^51^V, and ^66^Zn were analyzed in KED mode using a He flow rate between 3 and 4 cm^3^ min^−1^, while ^107^Ag, ^9^Be, ^209^Bi, ^7^Li, ^202^Hg, ^98^Mo, ^208^Pb, and ^205^Tl were analyzed in normal mode. The optimized instrumental parameters and the elemental settings used for each analyte are reported in Table S3.

The matrix effect was determined comparing the slopes of the calibration function obtained in the absence (2% HNO_3_ *v/v* aqueous solutions) or presence (digested honey samples spiked with known amounts of analyte) of the matrix [70]. The data obtained accounted for a substantial absence of any matrix effect for all the analytes considered in the whole calibration range, hence quantification has been accomplished by means of external calibration. The samples, blank-diluted when necessary to lead analyte concentrations within the relevant calibration range, were analyzed in duplicate. Data obtained as a result of a triplicate ICP-MS measurement, were blank-corrected. To compensate for any signal instability, a solution of Rh (10 μg dm^−3^) was used as an internal standard, while the reliability of the measurements was ensured by analyzing one blank and a standard solution containing each analyte (50 μg dm^−3^) every 10 samples. Memory effects between consecutive samples were eliminated by interposing a washing cycle of 60 s with a 2% HNO_3_ *v/v* aqueous solution.

### 3.5. Optimization of the Composition of the Acidic/Oxidizing Mixture

After some preliminary evaluations, the digestion program (Table 1; pression, temperature and time) and water volume of the oxidizing mixture (4 cm^3^) were kept constant, while the factors considered in the design were the sample amount (0.5–1.0 g), and the ratio between the volumes of HNO_3_ and H_2_O_2_ (0.5/3 cm^3^–2/1.5 cm^3^). As a result, a two-level 2^2^ full factorial design was applied to improve the composition of the oxidizing mixture and minimize the dissolved organic carbon in digested sample.

To estimate the experimental error and validate the regression model, a duplicate of the central point was added to the factorial design. The responses of the design were the residual acidity in the digested sample and the efficiency of organic matter decomposition [71] (EOMD%), which was calculated with Equation (1):

EOMD% = [(TOC − DOC)/TOC]%(1)

where TOC is the total organic carbon, in mg kg^−1^; DOC is the dissolved organic carbon, in mg kg^−1^.

The experimental matrix obtained with the two-level full factorial design is reported in Table 7 as well as the experimental plan.

The results obtained with the full factorial design are reported in Table 7. Multilinear regression (MLR) provided the coefficients for Equation (2) for both the responses, residual acidity (*Y*_1_) and EOMD% (*Y*_2_), respectively, which are reported in Table 8.

*Y_n_* = *b*_0_ + *b*_1 × 1_ + *b*_2 × 2_ + *b*_12 × 1 × 2_ (2)

where *b*_0_ is a constant, *b*_1_ and *b*_2_ are the coefficients of the main effects of the factors *X*_1_ and *X*_2_, whereas *b*_12_ is the coefficient of their interaction.

As reported in Table 8, all the coefficients were significant for both the responses, including the coefficient of the interactions (*b*_12_) especially for EOMD%. From the experiments at the central point, the experimental error can be estimated, and the standard deviation for *Y*_1_ and *Y*_2_ were 0.06 and 0.42, respectively. The predicted values for the residual acidity and for EOMD% at the center point were 0.5 ± 0.1 and 96 ± 1. Since the difference values between the experimental and predicted values are not significant (*t*-test, α = 0.05), the model can be accepted.

**Figure 4:**
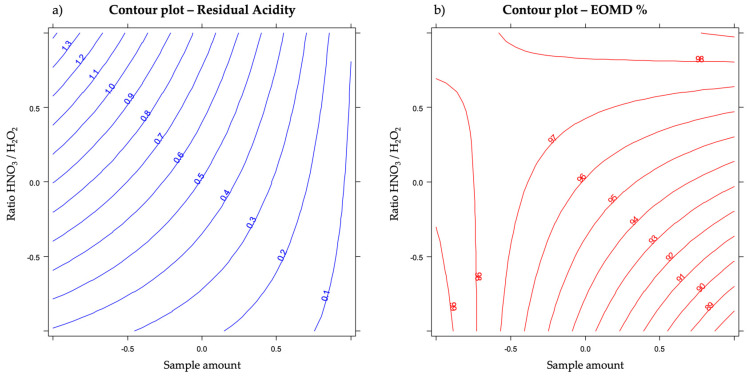
Contour plots for the effects on the design’s responses: (**a**) residual acidity; (**b**) EOMD%.

The effects of the sample amount and the ratio between the oxidizing compounds on the residual acidity and the EOMD% of the digested samples are shown in the contour plots in Figure 4.

From Figure 4 it is possible to understand the interaction between the two variables:

*Y*_1_. Residual Acidity: the effect of the ratio HNO_3_/H_2_O_2_ (*X*_2_) on the residual acidity is present only at the lower sample amount (*X*_1_) where its increase leads to higher acidity, while at higher amount var. *X*_2_ has no effect; on the other hand, at higher ratios (*X*_2_), an increase in the sample amount (*X*_1_) led to a higher decrease in the response, greater than the decrease occurring at lower ratios (*X*_2_).

*Y*_2_. EOMD%: the effect of the ratio HNO_3_/H_2_O_2_ (*X*_2_) on the EOMD is present only at the higher sample amount (*X*_1_) where its increase led to a higher response, while at the lower sample amount var. *X*_2_ had no effect; on the contrary, at lower ratios (*X*_2_), an increase in the sample amount (*X*_1_) led to a decrease in the response, while at higher ratios, the sample amount had no effect.

It is evident that a good compromise between the two responses provides a sample amount of approximately 0.7 g of honey and a ratio HNO_3_/H_2_O_2_ less than 0.5. Therefore, in order to minimize the consumption of nitric acid for the benefit of the greenest hydrogen peroxide, the acceptable composition of the acidic/oxidizing mixture is 0.5 cm^3^ HNO_3_, 3 cm^3^ of H_2_O_2_ and 4 cm^3^ of H_2_O.

### 3.6. Statistical Analysis

A two-tail *t*-test at α = 0.05 was used in ascertaining the existence of matrix effect as well as in the trueness evaluation. Principal components analysis, LDA, and MLR were performed by means of the R-based software Chemometric Agile Tool (CAT) developed by the Italian group of Chemometrics [72].

## 4. Conclusions

For the first time, the concentration of 23 trace elements were measured in a very large sampling of the most renowned unifloral honeys from Sardinia, Italy, using an original and validated ICP-MS method. Special attention was paid to the development of the acid microwave digestion procedure as well as the optimization of instrumental parameters to improve the efficiency of the organic matter decomposition and to minimize the polyatomic interferences, respectively. Among the most abundant elements (i.e., Ba, Mn, Fe, Cu, and Zn), only Mn was measured in all of the samples, whereas the others ranged from the relevant LoDs to a few mg kg^−1^. Toxic elements were almost always below the amounts of potential health concern, hence confirming a very good level of food safety for Sardinian honeys. Since no elemental signatures were reported in the literature for asphodel and thistle honeys, a meta-analysis was carried out only on eucalyptus and strawberry tree honeys and it highlighted the possibility of a geographical discrimination thanks to their elemental signature, mainly for strawberry tree honeys from Croatia and Sardinia. Finally, through the elemental signature of the four unifloral honeys here considered, a good classification based on the botanical origin was accomplished by means of linear discrimination analysis.

## Figures and Tables

**Table 1:** UltraWAVE SRC microwave digestion operational program and conditions.

Step		Time (min)	Temperature (°C)	
1st	Heating	25	240	Initial pressure: 40 bar Final temperature: <40 °C Pressure release rate: 8 bar/min Rack: 15 positions Vessel: volume 15 cm^3^, PTFE Sample amount: 0.7 g of honey Reagents: 0.5 cm^3^ HNO_3_, 3 cm^3^ of H_2_O_2_, and 4 cm^3^ H_2_O
2nd	Holding	10	240	
3rd	Cooling	~30	<40	

**Table 2:** Validation parameters of the ICP-MS method aimed at the determination of 23 trace elements in unifloral honeys.

Element	LoD ^a^	LoQ ^a^	Calibration Range ^b^	Repeatability ^c^	Intermediate Precision ^d^	Trueness,
	(μg kg^−1^)	(μg kg^−1^)	(μg dm^−3^)	CV (%)	CV (%)	Recovery (% ± s ^e^)
Ag	5	17	0.1–50	4	8	106 ± 5
As	2	7	0.1–50	4	6	92 ± 1
Ba	20	70	1–250	1	3	90 ± 20
Be	0.4	1.3	0.02–50	4	13	103 ± 1
Bi	0.1	0.3	0.005–50	4	12	85 ± 7
Cd	0.3	1.0	0.01–50	4	8	117 ± 1
Co	0.3	1.0	0.01–50	8	9	99 ± 1
Cr	7	23	0.1–50	4	5	97 ± 1
Cu	20	70	1–100	4	11	107 ± 4
Fe	30	100	1–100	4	17	105 ± 15
Hg	6	20	0.1–50	4	13	130 ± 10
Li	2	7	0.1–500	4	14	96 ± 1
Mn	8	27	0.1–500	3	3	107 ± 4
Mo	0.7	2.3	0.04–50	4	8	94 ± 1
Ni	3	10	0.1–100	6	18	95 ± 2
Pb	3	10	0.1–250	4	6	92 ± 1
Sb	0.7	2.3	0.04–50	4	9	115 ± 1
Sn	2.1	6.9	0.1–50	5	5	103 ± 2
Sr	3	10	0.1–100	3	7	103 ± 1
Te	1.2	3.9	0.04–50	4	21	108 ± 3
Tl	0.04	0.13	0.005–50	7	5	96 ± 1
V	0.2	0.7	0.01–50	4	6	94 ± 2
Zn	40	130	1–500	12	16	101 ± 1

^a^ The LoD and LoQ values were measured according to [58]. ^b^ Instrumental calibration range. ^c^ Evaluated on the sample replicates in the same analytical session (*n* = 3). ^d^ Evaluated on the sample replicates within one month (*n* = 3). ^e^ Standard deviation.

**Table 3:** Mean and range concentrations (in μg kg^−1^) of 23 toxic and trace elements in the unifloral honey samples of asphodel, eucalyptus, strawberry tree, and thistle.

Element	Asphodel (*n* = 33) *Asphodel* spp.		Eucalyptus (*n* = 30) *Eucalyptus* spp.		Strawberry Tree (*n* = 31) *Arbutus unedo* L.		Thistle (*n* = 39) *Galactites tomentosa*	
	Mean	Range	Mean	Range	Mean	Range	Mean	Range
Ag	*<5*	*<5*–<17	*<5*	*<5–<5*	*<5*	*<5*–<17	*<5*	*<5*–*<5*
As	<3	*<2*–9	<6	*<2*–19	*<2*	*<2*–<7	<4	*<2*–<7
Ba	180	*<20*–1600	340	80–690	550	<70–2600	300	<70–1800
Be	*<0.4*	*<0.4–<0.4*	*<0.4*	*<0.4–<0.4*	*<0.4*	*<0.4–<0.4*	*<0.4*	*<0.4–<0.4*
Bi	<0.2	*<0.1*–1.6	<0.2	*<0.1*–1.2	<0.2	*<0.1*–0.8	<0.6	*<0.1*–10.7
Cd	<0.7	*<0.3*–2.5	1.7	*<0.3*–9.2	<0.3	*<0.3*–<1	1.4	*<0.3*–5.2
Co	2.5	<1–10.2	7.6	<1–109	1.8	*<0.3*–9.7	5.1	<1–15.5
Cr	<11	*<7*–24	<17	*<7*–26	<15	*<7*–24	<8	*<7*–24
Cu	90	*<20*–250	170	<70–630	90	*<20*–250	180	<70–1030
Fe	160	<100–660	570	<100–1600	180	<100–630	340	120–820
Hg	*<6*	*<6–<6*	*<6*	*<6–<6*	*<6*	*<6*–<10	*<6*	*<6*–<20
Li	*<2*	*<2*–14	11	*<2*–30	280	*<2*–8500	<5	*<2*–22
Mn	190	40–770	2600	140–5100	330	<27–4900	330	40–3300
Mo	<1.8	*<0.7*–3.6	<2.3	*<0.7*–3.9	1.8	*<0.7*–3.8	<2.1	*<0.7*–3.7
Ni	19	*<3*–170	22	*<3*–122	12	*<3*–33	24	<10–220
Pb	23	*<3*–400	16	*<3*–95	<10	*<3*–90	<9	*<3*–30
Sb	<0.8	*<0.7*–<2.3	<1.3	*<0.7*–4.7	<0.9	*<0.7*–<2.3	<0.8	*<0.7*–2.8
Sn	44	*<2.1*–210	30	<6.9–110	43	*<2.1*–200	46	<7.1–240
Sr	38	*<3*–174	180	20–290	140	22–350	98	18–420
Te	<1.5	*<1.2*–6.8	*<1.2*	*<1.2–<1.2*	*<1.2*	*<1.2–<1.2*	*<1.2*	*<1.2*–<3.9
Tl	<0.13	*<0.04*–0.4	0.3	*<0.04*–2.2	<0.13	*<0.04*–1.4	0.18	*<0.04*–1.3
V	<0.4	*<0.2*–<0.7	4.1	<0.7–12.8	<0.6	*<0.2*–1.8	1.3	<0.7–5.6
Zn	550	<130–1400	660	330–1400	400	*<40*–1200	800	300–2000
Total (mg kg−1)	1.30	0.56–2.70	2.20	0.74–6.60	2.10	0.76–10.30	4.70	1.00–8.40

Each sample was analyzed twice. Italics represent data below the LoD; underlined values represent data below the LoQ.

**Table 4:** Mean concentration and range ^a^ (both in μg kg^−1^) of toxic and trace elements in unifloral Eucalyptus honeys from different geographical origins.

Element	Tunisia (*n* = 3) [36]	Argentina (*n* = 1) [37]	Italy (*n* = 1) [38]	Unknown (*n* = 1) [39]	Sardinia (*n* = 29) (This Work)
Ag					*<5*; *<5–<5*
As	19.08	<10	5.99	3.33	<6; *<2*–19
Ba					340; 80–690
Be		<10			*<0.4*; *<0.4–<0.4*
Bi					<0.2; *<0.1*–1.2
Cd	<0.01	<10	0.592	0.70	1.7; *<0.3*–9.2
Co		10			7.6; <1–109
Cr	130	<10	1.50	2.73	<17; *<7*–26
Cu	800	120	219	140	170; <70–630
Fe	7100	3380	1008	914	570; <100–1600
Hg			<0.75		*<6*; *<6–<6*
Li					11; *<2*–30
Mn	1250	8840	1009	1976	2600; 140–5100
Mo					<2.3; *<0.7*–3.9
Ni	220	50	11.3	8.04	22; *<3*–122
Pb	250	10	5.00	141	16; *<3*–95
Se	130	10	5.60		
Sb	100				<1.3; *<0.7*–4.7
Sn				7.85	30; <6.9–110
Sr					180; 20–290
Ti	610				
Te					*<1.2*; *<1.2–<1.2*
Tl		<10			0.3; *<0.04*–2.2
U		<10			
V	50	<10		3.36	4.1; <0.7–12.8
Zn	2060	550	791	414	660; 330–1400

^a^ Data are presented in the “average; range” format. *n* = number of honey samples analyzed. Italics represent data below the LoD; underlined values represent data below the LoQ.

**Table 5:** Results of the LDA, % of correct classified samples in the CV and prediction.

% Correctly Classified	Asphodel	Eucalyptus	Strawberry Tree	Thistle	Total
Cross- validation	61.9	84.2	78.9	76.9	75.5
Prediction ^a^	91.7	81.5	75.0	100.0	87.1

^a^ Internal validation.

**Table 6:** Confusion matrix in prediction.

	Asphodel	Eucalyptus	Strawberry Tree	Thistle
Asphodel	11	0	0	1
Eucalyptus	1	9	0	1
Strawberry tree	1	0	9	2
Thistle	0	0	0	13

**Table 7:** Two-level 2^2^ full factorial experimental design with center point.

Experiment	Sample Amount (g)	Ratio HNO_3_/H_2_O_2_	*X*_1_ ^a^	*X*_2_ ^a^	Residual Acidity (mol dm^−3^)	EOMD%
1	0.50	0.17	−1	−1	0.39	99.7
2	1.00	0.17	+1	−1	0.06	87.2
3	0.50	1.33	−1	+1	1.41	97.7
4	1.00	1.33	+1	+1	0.16	99.1
5	0.75	0.56	0	0	0.48	95.8
6	0.75	0.56	0	0	0.39	96.4

^a^ *X*_1_ and *X*_2_ represent the sample amount and the ratio HNO_3_/H_2_O_2_, respectively.

**Table 8:** Values of MLR coefficients and their significance levels for both the design’s responses.

	Residual Acidity		EOMD%	
Coefficient	Coefficient Value	Significance ^a^	Coefficient Value	Significance ^a^
*b*_0_	0.49	***	95.9	***
*b*_1_	−0.41	**	−2.8	*
*b*_2_	0.27	*	2.5	*
*b*_12_	−0.25	*	3.5	**

^a^ Blank, not statistically significant; * *p* < 0.05, ** *p* < 0.01, and *** *p* < 0.001.

## Data Availability

The data presented in this study are reported in the Supplementary Materials.

## Supplementary Materials

The following supporting information can be downloaded at: https://www.mdpi.com/article/10.3390/molecules27062009/s1, Figure S1: Scree plot; Figure S2: Influence plot; Figure S3: Permutation test. Table S1: Whole data set of the determination of 23 trace elements in 133 Sardinian unifloral honeys (Excel file); Table S2: Correlation coefficients; Table S3: Instrumental parameters and elemental settings used for the ICP-MS determination of 23 trace elements in unifloral honeys.

## Sample Availability

Honey samples are, in part, available from the authors.
