# Supplementary material for: Multi-Elemental Analysis as a Tool to Ascertain the Safety and the Origin of Beehive Products: Development, Validation, and Application of an ICP-MS Method on Four Unifloral Honeys Produced in Sardinia, Italy
Source: Molecules. 2022 Mar 21;27(6):2009. doi: 10.3390/molecules27062009 (PMC8950479; doi:10.3390/molecules27062009)
Supplement: Supplementary file 1 [file molecules-27-02009-s001.zip › Trace elements in Sardinian Honey_Supplementary material - Figures S1-S3, Table S2-S3.pdf]

Supplementary material

Figure S1. Scree plot of the PCA performed of the training set (85 samples, 18 variables)

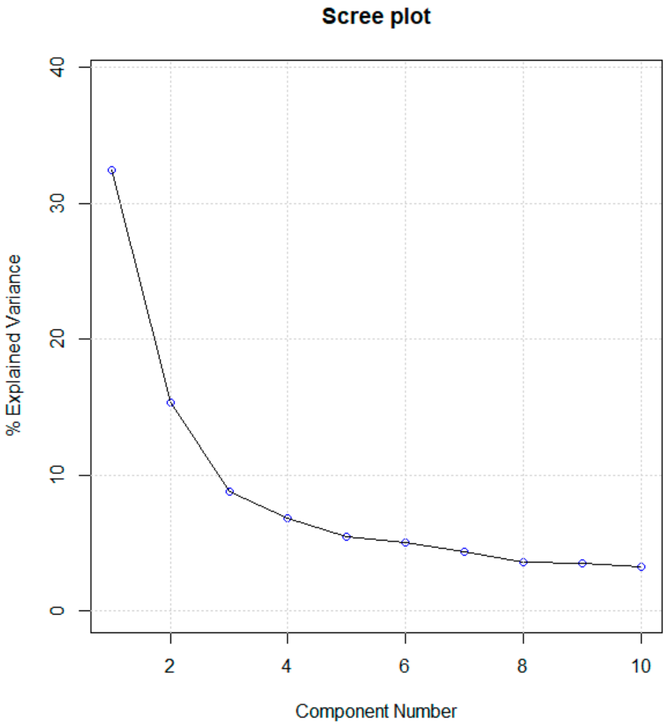

Figure S2. Influence plot of the PCA performed of the training set (85 samples, 18 variables)

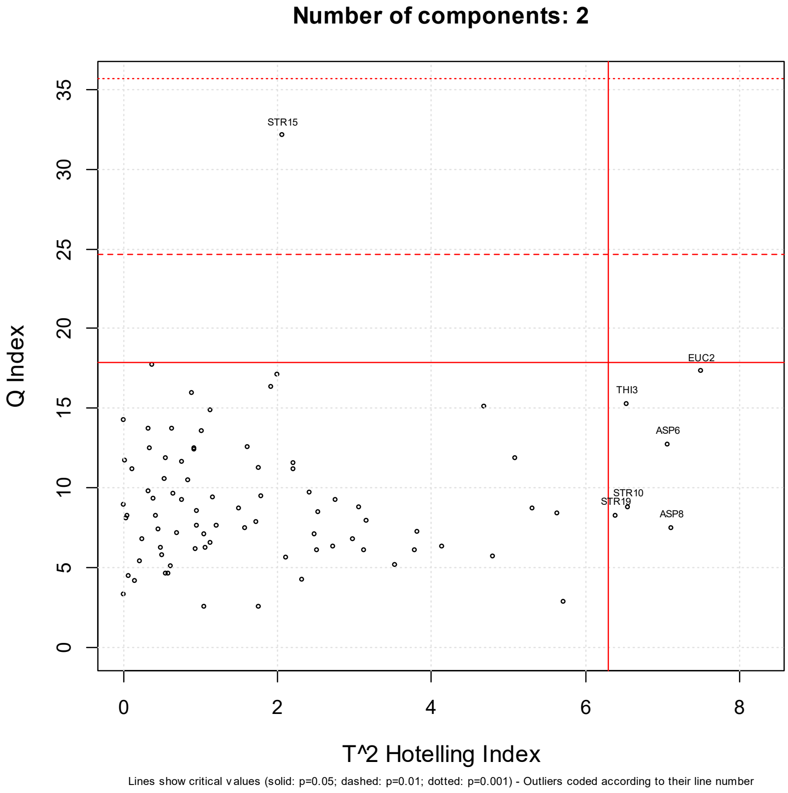

Figure S3. Permutation test of the LDA in calibration

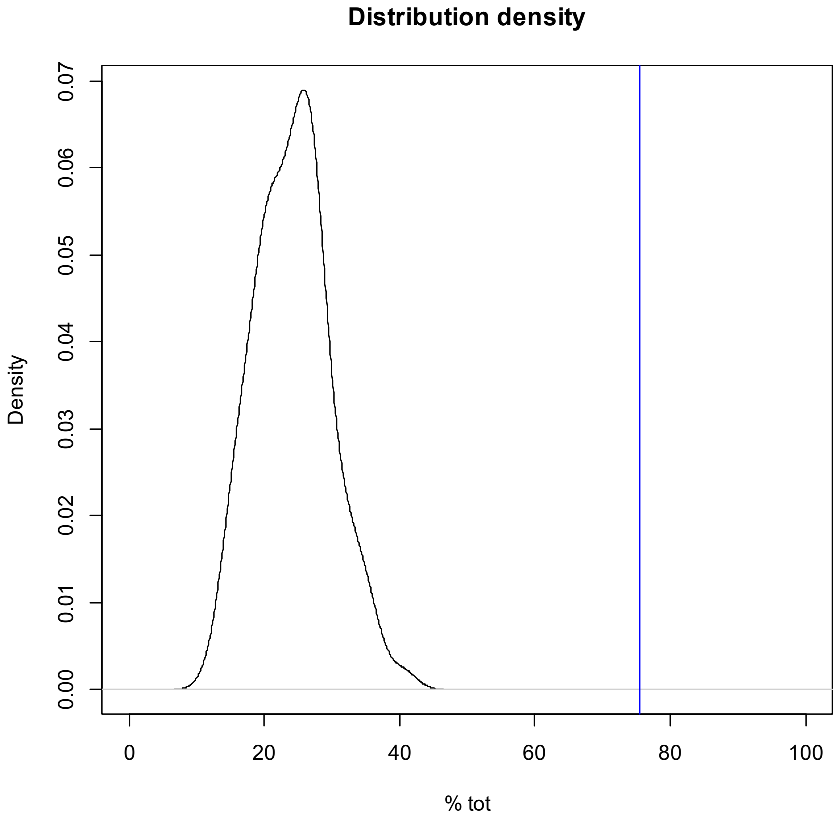

Table S2. Correlation's coefficients of the variables, before (blue) and after (red) the logarithmic transformation

	As	*Ba*	*Bi*	*Cd*	*Co*	*Cr*	*Cu*	*Fe*	*Li*	*Mn*	*Mo*	*Ni*	*Pb*	*Sn*	*Sr*	*Tl*	*V*	*Zn*
As	1.00	0.08	-0.05	0.26	0.02	-0.04	0.10	0.40	-0.04	0.34	0.29	-0.06	-0.01	0.01	0.29	0.08	0.40	0.16
Ba	0.22	1.00	-0.03	-0.05	-0.02	-0.04	-0.10	0.06	-0.04	0.05	0.07	-0.11	-0.09	0.11	0.37	0.13	0.10	-0.17
Bi	0.19	0.09	1.00	-0.03	0.05	-0.01	0.00	0.00	0.00	-0.01	0.01	-0.01	-0.04	-0.04	0.06	-0.04	-0.02	-0.01
Cd	0.44	-0.08	0.19	1.00	0.13	-0.01	0.30	0.33	-0.02	0.24	0.25	0.06	0.03	-0.04	0.13	0.37	0.33	0.56
Co	0.36	0.09	0.33	0.63	1.00	0.22	0.35	0.27	-0.03	0.27	0.19	0.19	-0.03	-0.08	0.18	0.25	0.34	0.30
Cr	0.02	0.03	0.19	0.18	0.24	1.00	0.00	0.17	-0.01	0.13	0.18	0.16	-0.02	0.04	0.12	0.02	0.17	0.04
Cu	0.23	0.01	0.21	0.52	0.68	0.01	1.00	0.37	0.05	0.21	0.23	0.75	-0.05	-0.05	0.04	0.25	0.22	0.62
Fe	0.49	0.32	0.43	0.48	0.62	0.24	0.60	1.00	0.07	0.72	0.51	0.15	-0.08	0.01	0.56	0.34	0.88	0.28
Li	0.24	0.39	0.23	0.13	0.13	0.13	0.16	0.49	1.00	-0.03	0.10	-0.01	-0.01	-0.08	0.05	0.01	0.02	-0.02
Mn	0.43	0.35	0.26	0.35	0.52	0.14	0.49	0.75	0.51	1.00	0.28	0.03	-0.04	-0.15	0.59	0.15	0.78	0.09
Mo	0.30	0.23	0.22	0.36	0.40	0.23	0.42	0.56	0.21	0.39	1.00	0.04	-0.07	0.11	0.28	0.19	0.49	0.25
Ni	0.05	-0.13	0.22	0.35	0.52	0.23	0.59	0.37	0.00	0.22	0.25	1.00	0.00	-0.01	-0.11	0.12	0.03	0.35
Pb	0.23	0.10	-0.06	0.23	0.14	0.01	0.16	0.20	0.02	0.14	0.10	0.17	1.00	0.23	-0.11	0.11	-0.03	0.05
Sn	0.14	0.12	-0.01	-0.01	0.00	-0.05	0.02	0.06	-0.11	-0.10	0.14	0.12	0.25	1.00	0.04	0.07	-0.09	0.11
Sr	0.35	0.72	0.28	0.11	0.26	0.17	0.15	0.56	0.61	0.55	0.29	-0.06	0.13	-0.03	1.00	0.08	0.59	-0.01
Tl	0.21	-0.01	0.19	0.44	0.47	0.13	0.27	0.23	0.01	0.08	0.13	0.23	0.23	0.07	0.03	1.00	0.37	0.33
V	0.47	0.29	0.31	0.47	0.60	0.30	0.47	0.84	0.48	0.76	0.56	0.19	0.21	-0.03	0.54	0.20	1.00	0.18
Zn	0.24	-0.17	0.25	0.60	0.62	0.16	0.61	0.45	0.12	0.32	0.34	0.46	0.09	0.07	0.06	0.41	0.41	1.00

Table S3. Instrumental parameters and elemental settings used for the ICP-MS determination of 23 trace elements in unifloral honeys.

**ICP-MS NexION 300X Perkin Elmer settings**			
**RF power generator (W)**	1300	**KED mode cell entrance voltage (V)**	-8.0
**Ar plasma flow (dm^3 min^{-1})**	18.0	**KED mode cell exit voltage (V)**	-25.0
**Ar auxiliary flow (dm^3 min^{-1})**	1.20	**Resolution (Da)**	0.7
**Ar nebulizer flow (dm^3 min^{-1})**	0.91	**Scan mode**	Peak hopping
**Nebulizer**	Meinhardt®, glass	**Detector mode**	Dual
**Spray chamber**	Cyclonic, glass	**Dwell time (ms)**	50
**Skimmer and sampling cones**	Nickel	**Number of points per peak**	3
**Sampling depth (mm)**	0	**Acquisition time (s)**	6
**Deflector voltage (V)**	-8.00	**Acquisition dead time (ns)**	35
**Analog stage voltage (V)**	-1750	**KED gas**	Helium 99.999%
**Pulse stage voltage (V)**	+1350	**Masses of optimization**	^{7}Li, ^{115}In and ^{205}Tl

**Quantification ion (% abundance)**	**Interfering ions**	**Analysing mode**	**He flow rate (cm^3 min^{-1})**	**Correction equation**
$^{107}Ag^+$ (51.84)	$^{91}Y^{16}O^+$, $^{91}Zr^{16}O^+$	Normal	-	
$^{75}As^+$ (100)	$^{40}Ar^{35}Cl^+$; $^{59}Co^{16}O^+$; $^{39}K^{36}Ar^+$; $^{63}Cu^{12}C^+$; $^{40}Ca^{35}Cl^+$;	KED	3.0	
$^{138}Ba^+$ (71.7)	$^{40}Ar_2{}^{58}Ni^+$; $^{138}La^+$; $^{122}Sn^{16}O^+$; $^{121}Sb^{16}O^1H^+$	KED	4.0	-0.000901x^{139}La -0.002838x^{140}Ce
$^{9}Be^+$ (100)	none	Normal	-	
$^{209}Bi^+$ (100)	none	Normal	-	
$^{111}Cd^+$ (12.80)	$^{95}Mo^{16}O^+$; $^{97}Mo^{14}N^+$; $^{79}Br^{16}O_2{}^+$; $^{94}Zr^{16}O^1H^+$; $^{71}Ga^{40}Ar^+$	KED	4.0	
$^{59}Co^+$ (100)	$^{24}Mg^{35}Cl^+$; $^{40}Ar^{18}O^1H^+$; $^{27}Al^{16}O_2{}^+$; $^{24}Mg^{35}Cl^+$	KED	3.5	
$^{52}Cr^+$ (83.79)	$^{40}Ar^{12}C^+$; $^{36}Ar^{16}O^+$; $^{1}H^{35}Cl^{16}O^+$; $^{40}Ca^{12}C^+$; $^{38}Ar^{14}N^+$	KED	3.0	
$^{63}Cu^+$ (69.17)	$^{40}Ar^{23}Na^+$; $^{31}P^{16}O_2{}^+$; $^{47}Ti^{16}O^+$; $^{28}Si^{35}Cl^+$; $^{51}V^{12}C^+$	KED	4.0	
$^{57}Fe^+$ (2.12)	$^{40}Ar^{16}O^1H^+$; $^{40}Ca^{16}O^1H^+$; $^{40}K^{16}O^1H^+$	KED	3.0	
$^{7}Li^+$ (92.50)	none	Normal	-	
$^{202}Hg^+$ (22.86)	$^{186}W^{16}O^+$	Normal	-	
$^{55}Mn^+$ (100)	$^{40}Ar^{14}N^1H^+$; $^{37}Cl^{18}O^+$; $^{39}K^{16}O^+$	KED	3.0	
$^{98}Mo^+$ (24.13)	$^{98}Ru^+$; $^{81}Br^{17}O^+$; $^{40}K_2{}^{18}O^+$; $^{58}Ni^{40}Ar^+$; $^{63}Cu^{35}Cl^+$	Normal	-	-0.10961 x ^{101}Ru
$^{60}Ni^+$ (26.22)	$^{44}Ca^{16}O^+$; $^{43}Ca^{16}O^1H^+$; $^{23}Na^{37}Cl^+$; $^{25}Mg^{35}Cl^+$; $^{28}Si^{16}O_2{}^+$	KED	3.5	
$^{208}Pb^+$ (52.40)	none	Normal	-	
$^{121}Sb^+$ (57.21)	$^{107}Ag^{14}N^+$; $^{109}Ag^{12}C^+$; $^{105}Pd^{16}O^+$; $^{81}Br^{40}Ar^+$; $^{120}Sn^1H^+$	KED	3.5	
$^{120}Sn^+$ (32.58)	$^{39}K^{81}Br^+$; $^{80}Se^{40}Ar^+$; $^{104}Pd^{16}O^+$; $^{104}Ru^{16}O^+$	KED	3.5	
$^{88}Sr^+$ (82.58)	$^{56}FeO_2{}^+$; $^{87}Rb^1H^+$; $^{48}Ti^{40}Ar^+$	KED	3	
$^{130}Te^+$ (34.08)	$^{95}MoO_2{}^+$; $^{114}Cd^{16}O^+$; $^{90}Zr^{40}Ar^+$	KED	4	-0.0094 x ^{137}Ba – 0.1543 x ^{129}Xe
$^{205}Tl^+$ (70.26)	$^{189}Os^{16}O^+$	Normal	-	
$^{51}V^+$ (99.75)	$^{35}Cl^{16}O^+$	KED	3.0	
$^{66}Zn^+$ (27.90)	$^{50}Ti^{16}O^+$; $^{65}Cu^1H^+$; $^{26}Mg^{40}Ar^+$; $^{31}P^{35}Cl^+$; $^{52}Cr^{14}N^+$	KED	3.0	
